# Psychographic Segmentation: Another Lever for Precision Population Brain Health

**DOI:** 10.3389/fnagi.2021.783297

**Published:** 2021-12-08

**Authors:** Erin Smith, Agustin Ibanez, Helen Lavretsky, Michael Berk, Harris A. Eyre

**Affiliations:** ^1^Neuroscience-Inspired Policy Initiative, Organization for Economic Co-operation and Development (OECD) and the PRODEO Institute, Paris, France; ^2^Global Brain Health Institute, University of California, San Francisco (UCSF), San Francisco, CA, United States; ^3^Trinity College Dublin, Dublin, Ireland; ^4^Department of Medicine, Stanford Hospital, Stanford, CA, United States; ^5^The PRODEO Institute, San Francisco, CA, United States; ^6^Cognitive Neuroscience Center (CNC), Universidad de San Andrés, Buenos Aires, Argentina; ^7^Latin American Brain Health (BrainLat), Universidad Adolfo Ibáñez, Santiago, Chile; ^8^National Scientific and Technical Research Council (CONICET), Buenos Aires, Argentina; ^9^Semel Institute for Neuroscience and Human Behavior, University of California, Los Angeles (UCLA), Los Angeles, CA, United States; ^10^The Institute for Mental and Physical Health and Clinical Translation (IMPACT), Deakin University, Geelong, VIC, Australia; ^11^Department of Psychiatry, University of Melbourne, Melbourne, VIC, Australia; ^12^Orygen Youth Health, University of Melbourne, Melbourne, VIC, Australia; ^13^The Florey Institute for Neuroscience and Mental Health, University of Melbourne, Melbourne, VIC, Australia; ^14^Department of Psychiatry and Behavioral Sciences, Baylor College of Medicine, Houston, TX, United States

**Keywords:** psychographic segmentation, precision/personalized medicine, population health, dementia prevention, patient engagement

## Abstract

Dementia prevention interventions that address modifiable risk factors for dementia require extensive lifestyle and behavior changes. Strategies are needed to enhance engagement and personalization of the experience at a population level. Precision Population Brain Health aims to improve brain health across the lifespan at a population level. Psychographic segmentation is a core component of Precision Population Brain Health with untapped potential. Psychographic segmentation applies behavioral and social sciences to understanding people’s motivations, values, priorities, decision making, lifestyles, personalities, communication preferences, attitudes, and beliefs. Integrating psychographic segmentation into dementia care could provide a more personalized care experience and increased patient engagement, leading to improved health outcomes and reduced costs. Psychographic segmentation can enhance patient engagement for dementia and shift the clinical paradigm from “What is the matter?” to “What matters to you?” Similar benefits of psychographic segmentation can be provided for dementia caregivers. Developing dementia prevention programs that integrate psychographic segmentation could become the basis for creating a shared framework for prevention of non-communicable diseases and brain health disorders at a population level. Integrating psychographic segmentation into digital health tools for dementia prevention programs is especially critical to overcome current suboptimal approaches. Applying psychographic segmentation to dementia prevention has the potential to help people feel a sense of empowerment over their health and improve satisfaction with their health experience—creating a culture shift in the way brain health is approached and paving the way toward Precision Population Brain Health.

## Introduction

The dearth of clinically established dementia therapeutics emphasizes the importance of prevention approaches (Rabinovici, 2021). The 2020 Lancet Commission on dementia prevention, intervention, and care identified 12 modifiable risk factors for dementia and developed a life-course model of dementia prevention based on these factors (Livingston et al., 2020). The risk factors are less education, hypertension, hearing impairment, smoking, obesity, depression, physical inactivity, diabetes, low social contact, excessive alcohol consumption, traumatic brain injury (TBI), and air pollution (Livingston et al., 2020). Modifying these 12 risk factors might prevent or delay up to 40% of dementias and maybe even more in low-income and middle-income countries where around two-thirds of people with dementia live (Livingston et al., 2020; Patterson, 2018). Dementia prevention interventions that address modifiable risk factors for dementia, such as the Finnish geriatric intervention study to prevent cognitive impairment and disability (FINGER) (Ngandu et al., 2015), require extensive lifestyle and behavior changes. Consequently, strategies are needed to enhance engagement and personalization of the experience at a population level.

Precision Population Brain Health aims to improve brain health across the lifespan at a population level. It fuses Precision Brain Health (Fernandes et al., 2017; Frisoni et al., 2020) and Population Brain Health (UCSF Center for Population Brain Health, 2021). Engagement and personalization at a population level is key to Precision Population Brain Health. For Precision Population Brain Health to be achieved, insights from various disciplines must be combined. For example, for efforts such as widespread dementia prevention, platform technologies (e.g., telemedicine, apps) and frontier technologies (e.g., genomics, AI, robotics), creative care, culturally competent care, personalization techniques, and behavior design must be combined to address social determinants and other modifiable risk factors for dementia.

Psychographic segmentation is a core component of Precision Population Brain Health with untapped potential. Psychographic segmentation applies behavioral and social sciences to understanding people’s motivations, values, priorities, decision making, lifestyles, personalities, communication preferences, attitudes, and beliefs (Samuel, 2016). Complementing demographic and socioeconomic segmentation, psychographic segmentation enables people to be divided into sub-groups based on shared psychological characteristics. Since the 1970s, psychographic segmentation has been used by the world’s most successful consumer product and retail companies to understand and influence consumer behavior. It is commonly leveraged by the corporate sector to better understand employees and build more successful organizations. Psychographic segmentation has potential to improve dementia prevention initiatives and to provide a more ecological, decision making-oriented evaluation of both people with dementia and their caregivers.

## Psychographic Segmentation for Health

Psychographic segmentation has only recently started to be used for healthcare applications. Currently, many healthcare programs are one-size-fits-all or are based on a shared diagnosis. This often does not lead to high adoption rates of recommended behaviors. This highlights a need for personalization, creating an opportunity for psychographic segmentation to increase patient engagement and improve outcomes (Hardcastle and Hagger, 2015). Applying psychographic segmentation to healthcare enables organizations within healthcare to understand—and classify accordingly—if individual consumers take a proactive or reactive approach to health and wellness; want many or few choices; want traditional and/or alternative medicine; prioritize others’ health and wellness over their own; and more. For example, if someone lives in the “here and now” and does not prioritize their long-term health, messages from a health system may be sent *via* text instead of email and include language that emphasizes living for today, clear first steps, and immediacy. Companies including Frame Health and PatientBond have been key to actioning and scaling psychographic segmentation within healthcare, including capturing psychographic data and integrating it into electronic health records (EMRs) and customer relationship management platforms (CRMs) (Frame Health, 2021; PatientBond, 2021a). In partnership with Frame Health for example, CVS, UnitedHealthcare, and Cedars-Sinai Medical Center were able to increase patient signups for a medication adherence program by 38% and decrease pharmacy all time by 22% using Frame Health’s personalized call strategies and scripts (Frame Health, 2020). As another example, in partnership with PatientBond, a top health system was able to reduce hospital readmissions for congestive heart failure by 90% using PatientBond’s digital system (PatientBond, 2020).

However, the use of psychographic segmentation for dementia—along with brain health more broadly—remains nascent. Integrating psychographic segmentation into dementia care could provide a more personalized care experience and increased patient engagement, leading to improved health outcomes and reduced costs (Laurance et al., 2014). Psychographic segmentation has the opportunity to enhance patient engagement for dementia and to “change the clinical paradigm from ‘What is the matter?’ to ‘What matters to you?”’ (Edgman-Levitan et al., 2013).

## Psychographic Segmentation for Dementia Prevention

Due to the complex, multifactorial, and heterogeneous nature of dementia, there has been increasing interest in multidomain interventions for dementia prevention (Andrieu et al., 2015). Multidomain interventions target several risk factors and mechanisms simultaneously and may be required for optimal preventative effects (Rosenberg et al., 2020b). The first large-scale randomized controlled trials (RCTs) of multidomain interventions include the Finnish Geriatric Intervention Study to Prevent Cognitive Impairment and Disability (FINGER) trial and the Multidomain Alzheimer Preventive Trial (MAPT).

In FINGER, participants received a 2-year multidomain lifestyle intervention consisting of physical training, cognitive training, nutritional counseling, and cardiovascular monitoring (Kivipelto et al., 2013; Ngandu et al., 2015). In MAPT, participants received a 3-year multidomain lifestyle intervention consisting of cognitive training, physical activity counseling, and nutritional counseling with either an omega-3 supplement or placebo (Andrieu et al., 2017). For multidomain interventions such as FINGER and MAPT, adherence is key (Coley et al., 2019). Greater personalization, such as considering participant characteristics and motivations and taking a precision prevention approach, is critical to increase adherence (Coley et al., 2019; Rosenberg et al., 2020a; Solomon et al., 2021). In other words, a psychographic segmentation approach to prevention is needed.

**FIGURE 1:**
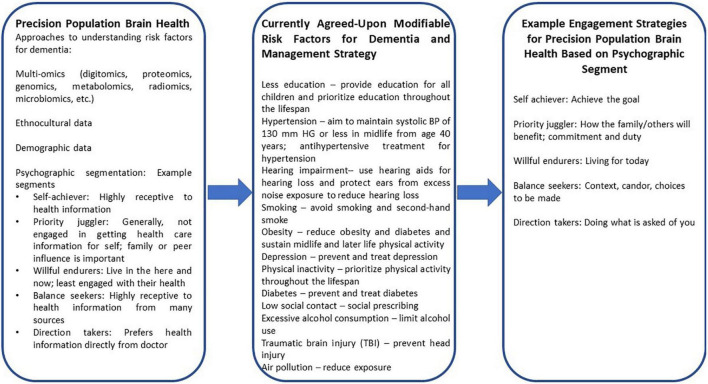
Examples of how psychographic segmentation can enable a Precision Population Brain Health approach to care through addressing modifiable risk factors of dementia and enhancing patient engagement.

Psychographic segmentation has the potential to improve the adherence and effectiveness of multidomain interventions for dementia prevention. For instance, a few example segments of psychographic segmentation include the following (see Figure 1 for more) (PatientBond, 2021b): (1) Self-achiever, people who are highly receptive to health information; (2) priority juggler, people who are generally not engaged in getting health care information for themselves and whose family or peer influence is important; and (3) Willful endurers, people who live in the here and now and are least engaged with their health. The engagement strategies will need to be varied and personalized for each segment. For example, outreach materials and participation strategies for self-achievers may be centered around the theme of achieving a goal, whereas for priority jugglers, a sense of how the family/other people will benefit and evoking commitment and duty will be essential. For the willful endurers, messages that center around living for today and create a sense of urgency will be important. The method of communication may differ with certain segments preferring text messaging, emails, or physical mail. Different versions of dementia preventions programs could be developed for different psychographic profiles. Since dementia prevention requires multifactorial efforts across the lifespan, these methods of enhancing engagement and personalization through psychographic segmentation are key and a powerful addition to researchers’ and health providers’ toolkits.

## Psychographic Segmentation for Digital Technologies for Dementia

Both in-person and digital dementia prevention programs could extensively benefit by integrating psychometric segmentation into their approach. With the proliferation of digital health tools for dementia prevention programs, it is especially important to integrate psychographic segmentation into these technologies. When describing hallmarks of digital health initiatives that have not lived up to their potential, Dr. Brennan Spiegel, Director of Health Services Research at Cedars-Sinai Health System, points toward not giving patients optimal messaging, not inviting patients in the most compelling way, and a lack of patient engagement as being problematic (Fry and Mukherjee, 2018) – all of which could have been helped by psychographic segmentation. He further noted that with digital health tools “Creating the tech isn’t the hard part…the hard part is using the tech to change patient behavior” (Pagoto and Hekler, 2018). Psychometric segmentation is highly compatible with current applications of behavioral insights across the health system, especially for nudging (Hansen et al., 2016; Benartzi et al., 2017). Such interventions can be boosted by the development behavioral research in policy domains to increase effectiveness, economic growth, and competitiveness (Sunstein, 2016). The combination of technology and psychometry are a powerful force to change behavior that should be ethically addressed to avoid technocracy and “psychocracy” (Feitsma, 2018). Integrating psychometric segmentation into digital health tools for dementia may help address these challenges.

## Psychographic Segmentation for Caregivers

Psychographic segmentation can also improve the lives of caregivers for people with dementia. For example, a recent study explored how people balance caregiving with other family and employment responsibilities by considering their personal characteristics and their informal caregiving network (Neubert et al., 2018). Applying psychographic segmentation could expand this study and lead to a more comprehensive understanding of a caregiver’s needs. Additionally, better understanding of a caregiver’s motivations and personality through psychographic segmentation can be used to develop personalized strategies to mitigate caregiver burden and prevent burnout.

## Conclusion

Applying psychometric segmentation to dementia prevention endeavors can improve the entire spectrum of care. Psychometric segmentation accounts for an individual’s unique values, motivations, priorities, lifestyle, personality, and beliefs as well as behavioral change. Thus, personalization and patient engagement can be at the heart of care. In addition to improved health outcomes and reduced costs due to dementia, developing dementia prevention programs that integrate psychographic segmentation could become the basis for creating a shared framework for prevention of non-communicable diseases and brain health disorders at a population level (O’Neil et al., 2015). Applying psychometric segmentation to dementia prevention endeavors has the potential to help people feel a sense of empowerment over their health and improve satisfaction with their health experience—creating a culture shift in the way brain health is approached and paving the way toward Precision Population Brain Health.

## Data Availability Statement

The original contributions presented in the study are included in the article/supplementary material, further inquiries can be directed to the corresponding author/s.

## Author Contributions

ES led the manuscript development. ES and HE co-developed the idea that led to the beginning of this manuscript. All authors contributed to the idea development, writing, and editing of this manuscript and approved the submitted version.

## Conflict of Interest

The authors declare that the research was conducted in the absence of any commercial or financial relationships that could be construed as a potential conflict of interest.

## Publisher’s Note

All claims expressed in this article are solely those of the authors and do not necessarily represent those of their affiliated organizations, or those of the publisher, the editors and the reviewers. Any product that may be evaluated in this article, or claim that may be made by its manufacturer, is not guaranteed or endorsed by the publisher.
